# Biomarkers for Diagnosis, Prognosis and Response to Immunotherapy in Melanoma

**DOI:** 10.3390/cancers13122875

**Published:** 2021-06-09

**Authors:** Rossella Puglisi, Maria Bellenghi, Giada Pontecorvi, Giulia Pallante, Alessandra Carè, Gianfranco Mattia

**Affiliations:** Center for Gender-Specific Medicine, Istituto Superiore di Sanità, Viale Regina Elena 299, 00161 Rome, Italy; rossella.puglisi@iss.it (R.P.); maria.bellenghi@iss.it (M.B.); giada.pontecorvi@iss.it (G.P.); giulia.pallante@iss.it (G.P.); gianfranco.mattia@iss.it (G.M.)

**Keywords:** melanoma, biomarkers, immunotherapy, BRAF, NRAS

## Abstract

**Simple Summary:**

Cutaneous Melanoma is a form of skin cancer characterized by an elevated mutational load that favors high spread to distant organs and resistance to therapies. The identification of biomarkers, able to dissect normal and pathogenic biological processes and response to therapeutic intervention, is necessary to describe melanoma as accurately as possible, having a positive impact on early diagnosis, in turn selecting the best therapeutic option. Recently, a great number of new biomarkers were evaluated, in order to identify those patients who may have clinical benefit from a therapeutic choice, particularly for immunotherapy. At present, these new biomarkers wait to be validated before clinical use. Hence, the requirement to look at and periodically update the advances in this field.

**Abstract:**

Cutaneous Melanoma classification is constantly looking for specific and sensitive biomarkers capable of having a positive effect on diagnosis, prognosis and risk assessment, eventually affecting clinical outcome. Classical morphological, immunohistochemical and the well-known BRAF and NRAS genetic biomarkers do not allow the correct categorization of patients, being melanoma conditioned by high genetic heterogeneity. At the same time, classic prognostic methods are unsatisfactory. Therefore, new advances in omics and high-throughput analytical techniques have enabled the identification of numerous possible biomarkers, but their potentiality needs to be validated and standardized in prospective studies. Melanoma is considered an immunogenic tumor, being the first form of cancer to take advantage of the clinical use of the immune-checkpoint blockers. However, as immunotherapy is effective only in a limited number of patients, biomarkers associated with different responses are essential to select the more promising therapeutic approach and maximize clinical benefits. In this review, we summarize the most utilized biomarkers for Cutaneous Melanoma diagnosis, focusing on new prognostic and predictive biomarkers mainly associated with immunotherapy.

## 1. Introduction

In the last decade, research in melanoma treatment saw two historical moments, defined in 2011 by the FDA approval of ipilimumab and vemurafenib for BRAF proto-oncogene (BRAF) mutated metastatic melanoma treatment, and in 2014 by the authorization of pembrolizumab and nivolumab for unresectable and metastatic melanoma. These two different approaches, targeted therapy and immunotherapy, are able to attack the advanced stages of melanoma that, until those dates, accounted for the majority of skin cancer related death, although representing less than 5% of all cutaneous malignancies [1].

This new picture in the control of advanced melanoma, even if representing a milestone in cancer treatment, still evidences some dark zones, since patients suffer from drug resistance or are non-responders, and progression free or overall survival are still low [2]. Certainly, we need to increase knowledge of the molecular pathways responsible for melanoma pathogenesis and progression, but also understand why effectiveness of therapies is so different among patients. Just after a full comprehension of the underlying specificities, we will be able to select the more promising therapeutic approach recognizing the risk of disease progression or the actual chance of response. 

At diagnosis, 85% of patients have circumscribed disease, 15% show regional lymph nodal involvement, about 2–5% present distant metastases [3]. Determination of melanoma staging results from a complex histopathological examination of lesions that also considers the absence of specific and sensitive biomarkers with exclusive features and that is capable of obtaining univocal impact for prognosis assessment and treatment planning.

The American Joint Committee on Cancer (AJCC) has recently revised melanoma classification based on Breslow thickness. In this implementation, patients can be stratified in risk category by the TNM (tumor, lymph node, metastasis) system, individuating four stages, stage I–IV, comprising different localizations of the disease with different impact on the overall survive and profound effects on prognosis. Stage I and II identify localized disease, while stage III and IV diagnose disease progression with nodal or distant metastases respectively. In AJCC classification, primary tumors are also divided in four classes (T1–T4) based of tumor thickness, again subdivided in a or b for absence or presence of ulcerations. Nodal category indicates whether melanoma has moved from the primary site to the vicinal lymph nodes and is individuated by different subgroups, designated by 0 to 3, indicating the absence (N0) or presence of lymph nodal metastasis. With M are classified only melanoma that have developed distant metastases and represent the most advanced staging of disease [4]. In this context, the accuracy and improvement of melanoma diagnosis, staging and risk assessment are essential for adequate prognostication, eventually allowing for the best therapeutic choice. Classical diagnostic and prognostic methods have evidenced accuracy failure in patient stratification. However, if we look at the last 20 years, the evolution of melanoma biomarkers discovery went from around 900 biomarker publications in 1999 to 51,000 in 2019 [5]. Indeed, the recent combination with new omics technologies from tumor or sera of patients, have produced an impressive increment of new possible biomarkers. However, all these potential biomarkers need to be investigated in large-scale studies for validation and standardization.

This review provides information on the current landscape of biomarkers for melanoma diagnosis, prognosis and prediction described in the literature, reporting those with the best impact on disease classification. In addition, in view of the recently developed immunotherapies, we also describe some biomarkers recently associated with different response to immune checkpoint blockers (ICBs) treatment. Indeed, biomarkers predicting clinical benefits are a new scientific challenge and their identification essential for an actual precision medicine.

## 2. Pathogenic and Epidemiological Diagnostic Biomarkers in Melanoma 

**Figure 1:**
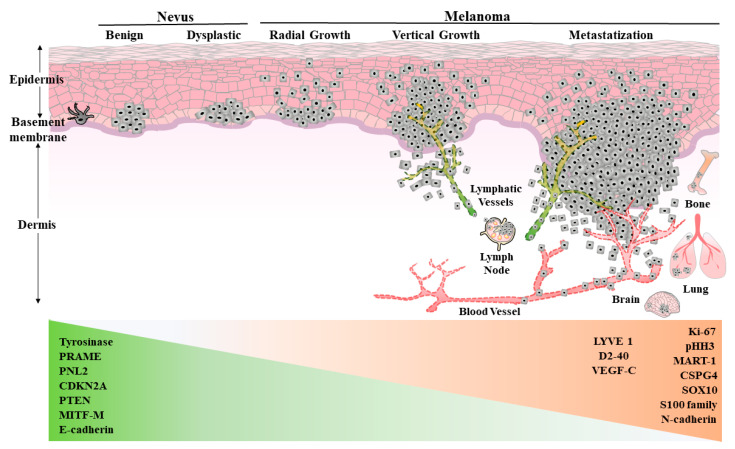
Schematic picture of melanoma progression from benign nevus to metastatic stage. Representative melanocytic associated and lymphangiogenic specific immunohistochemical markers are reported. PRAME: preferential expressed antigen in melanoma, CDKN2A: cyclin dependent kinase inhibitor 2A, PTEN: phosphatase and tensin homolog, MITF-M: microphthalmia transcription factor protein M, LYVE-1: lymphatic vessel endothelial hyaluronan receptor 1, D2-40: podoplanin antibody, VEGF-C: vascular endothelial growth factors C, pHH3: phospho-Histone H3, MART-1: melanoma antigen recognized by T cells 1, CSPG4: chondroitin sulfate proteoglycan 4, SOX10: SRY-related HMG-box transcription factor 10.

Cutaneous Melanoma (CM) is characterized by extensive mutational burden causing elevated genetic heterogeneity, resistance to treatments and high propensity to spread. Therefore, the necessity to individuate different therapeutic intervention and, more important, the need for early detection and diagnosis is essential. Briefly, melanoma cells originate either from normal epidermal melanocytes or from melanocytic cells internal to preexisting nevi (generally individuating a dysplastic condition). In both cases, some cells, losing growth control by multiple mutational hits, tend to accumulate in the epidermal stratum of the skin. This phase of melanoma growth is termed radial phase and tumor growth is circumscribed in the epidermal area (Figure 1). Then, its growth becomes vertical, when cells acquire the propensity to growth in the derma below. During transition from radial to vertical growth phase, melanoma cells begin to secrete angiogenic factors responsible for development of intratumor blood and lymphatic vessels. Dissemination to regional lymph nodes is usually the first metastatization event, suggesting melanoma cells propensity to take the road of the lymphatic vasculature. At the onset of this phase, melanoma cells undergo morphological and functional modifications through the epithelial mesenchymal transition (EMT) necessary to exalt their migratory capability, eventually allowing cell transmigration through the circulation or lymphatic vessels and then to metastasize in both regional lymph nodes and distant organs [6].

For a long time surgical resection of localized primary early lesions has been the only approach to counteract melanoma aggressiveness, with about 90% of patients getting to long-term survival. Conversely, advanced forms were mostly incurable.

In the last decade, an increment of incidence of CM was observed worldwide, particularly in aged subjects of more than fifty years. Fortunately, this increment is counterbalanced by the recent improvement in therapeutic approaches associated with target therapy and the promising immune checkpoint blockers, capable to significantly reduce mortality [7].

Recent data also evidenced that incidence rate and mortality of melanoma are influenced by gender disparity [8] and that melanoma incidence is growing faster in men than in women [9]. Ultraviolet exposure and behavioral difference in sun exposure and tanning between sexes play a role. These differences are made more complex by the less propensity of males to engage in preventive behaviors, also in consideration of the different body-site distribution, being melanoma more often on the lower extremities in females and more truncal in males, so in part detectable only in dermatological visit. These differences in early detection and primary care access can partly explain the female better survival [10,11]. Differences in survival between the two sexes have been associated with a lower tumor dissemination, explained by a different mutational burden in the female compared to male population, but also and more importantly with the more efficient female immune system [12,13].

Since melanoma classification remains a diagnostic challenge for the lack of the necessary specificity, including distinction between malignant and benign lesions, as well as the different forms and stages of melanoma, the identification of different level of diagnostic markers is necessary to frame the disease as accurately as possible.

### 2.1. Classic Diagnostic Criteria and Markers

The earliest step of disease indication is visual, through dermoscopy, according to ABCDE criteria of asymmetry, border irregularity, color variation, diameter (>6 mm) and evolution. This criterion is supported by the histopathological examination of the lesion in the vertical plane from epidermis to derma to individuate morphological characters that photograph the instant features of the tumor possibly associate to specific functionality and stage. At this level, cases of misinterpretation have been observed indicating the necessity of implementation in diagnostic criteria. Particularly, specific biomarkers are required to distinguish benign from malignant lesions. Among them, the proliferative marker Ki-67, found at very low level in common nevi and present in 30% up to 100% of melanoma cells, and the Melanoma antigen recognized by T cells-cloned gene (MART-1) can be utilized as markers for the presence of melanoma cells in the lymph nodes (Figure 1). Several other markers have been identified, but all show some limitation in their diagnostic capability. Important examples are the proteins of the S100 family, reported to control different cellular activity, from proliferation to migration, calcium homeostasis and protein phosphorylation and utilized for specific diagnosis [14,15]. Chondroitin Sulfate Proteoglycan 4 (CSPG4) is a plasma membrane protein showing high specificity for metastatic melanoma [16] while tyrosinase, the enzyme involved in melanin synthesis, is a biomarker useful for diagnosis of primary melanoma [17]. Conversely, a PNL2 monoclonal antibody specifically against an unknown target and the microphthalmia transcription factor protein M (MITF-M) are useful in the immunohistochemical panels, but demonstrated low specificity against melanoma cells, being present in neutrophilic population and in other tumor cells respectively [18]. Seemingly, the melanocyte specific isoform of the Melanocortin 1 Receptor (MC1R), involved in pigmentation of melanocytes, is not specific for melanoma cells, being also expressed in neuronal cells [19]. The protein SRY-related HMG-box transcription factor 10 (SOX10) involved in the determination of embryonal cell fate, represents a sensitive biomarker for melanoma and lymph nodal staining [20], whereas the preferential expressed antigen in melanoma (PRAME), p16 and phospho-histone H3 (pHH3) are utilized for specific staining panels for discrimination of uncertain lesions [21].

### 2.2. Tumor Specific Lymphatic Vessel Biomarkers

Lymphovascular invasion (LVI) has been shown in primary vertical growth phase melanomas, often associated with a positive sentinel lymph node biopsy and a worse clinical outcome [22] but with discussed predictive potential [23]. Until the discovery of some lymphatic endothelium specific antibodies, distinguishing blood vessels from lymphatics on patient histological specimens was a challenge for pathologists. Largely used in immunohistochemistry (IHC) were LYVE-1 (a lymphatic vessel endothelial hyaluronan receptor 1) [24] and D2-40 (a monoclonal antibody for podoplanin) [25], whereas the pan-endothelial cell markers CD31/CD34 were often used for blood vessel detection. In particular, double IHC assays combining these markers with others for melanoma, although not tumor-exclusive (S-100, MART-1, Melan A and MITF), were performed to better identify foci of lymphatic invasion in melanoma. Thus, improvement in histology field has enabled to better define LVI and evaluate the lymphatic vessel density (LVD) and localization, such as intra- or/and peritumoral, whose prognostic relevance remains to be fully determined [26].

### 2.3. Genetic Diagnostic Markers

Genetic diagnostic markers allow the best patient stratification also in view of therapeutic decision. Thanks to whole genome sequence approach, we were able to evidence how extraordinary the genetic heterogeneity in patients with melanoma is. The best-characterized mutation in melanoma, present in about 40–60% of patients, is in the BRAF proto-oncogene. More than 90% of these mutations harbor the transversion T to A, involving nucleotide 1799 and resulting in the substitution of valine by glutamic acid (V600E). More rare mutational events in the same codon involve lysine (V600K, 8–20%), arginine (*p*. V600R, 1%), methionine (*p*. V600M, 0.3%) or aspartic acid (*p*. V600D, 0.1%) [27]. BRAF mutations occur more frequently in young people, in no-chronical ultraviolet (UV) exposure and are more aggressive compared to wild type melanomas [28,29]. A second group of “driver” mutations involves the small GTPase Neuroblastoma rat sarcoma viral oncogene homolog (NRAS) proto-oncogene, representing 15–20% of all the BRAF-WT melanoma patients. Commonly, point mutations in NRAS lead to the substitution of glutamine to leucine at position 61, or rarely at positions 12 and 13 [30]. NRAS mutated tumors are aggressive with high mitotic activity and progression rate [31]. This group of patients is characterized by advanced age with chronic skin exposure to UV [32].

BRAF mutations are already present in benign nevi, but functional mechanisms of growth control for years limit their expansion in adjacent areas. Particularly in UV radiation-induced DNA damage, melanocytes respond with DNA repair or activation of senescence or apoptotic pathways. The mechanisms of DNA repair are notoriously error-prone and can contribute to accumulate mutations in DNA of melanocytes already carrying mutation of BRAF. Indeed, multiple mutational “hits” are necessary to transform a benign nevus into a melanoma in situ, being this event estimated to occur in a 0.03% and 0.009% between male and female, respectively, during the lifespan [33]. The picture is more problematic when genetic predisposition to the development of melanoma is genetically evident as in pathological conditions such as xeroderma pigmentosum (XP), congenital melanocytic nevi, familial atypical multiple moles and melanoma (FAMMM) syndrome, as well as breast cancer type 2 susceptibility protein (BRCA2) mutation [34,35]. Besides the influence of environmental factors, melanoma can occur in low sun exposed district (mucosal surfaces for instance), implicating that more than a linear mutational sequence can underlie melanoma development. However, considering the capability of eumelanin to protect DNA more efficiently than pheomelanin, a difference in incidence of melanoma was observed in lighter- than in darker-skinned individuals [36]. To schematize, going from nevus to melanoma is necessary the loss of cyclin dependent kinase inhibitor 2A (CDKN2A) and phosphatase and tensin homologue (PTEN), together with the down-regulation of MITF master gene in the evolution from radial to vertical growth phase melanoma. This characterizes dermal invasion of melanoma cells and involves the epithelial to mesenchymal transition gene modulation with loss of E-cadherin and increased expression of N-cadherin (Figure 1) [37]. Conversely, additional genetic mutations accumulating in NRAS mutated cells, mainly in chronically sun damaged skin, form intermediate dysplastic invasive lesions, generally associated to the tumor protein p53 (TP53) oncosuppressor gene silencing [38]. About 45% of melanomas, displaying wt BRAF and NRAS proteins, show mutation of the Neurofibromin 1 (NF1) oncosuppressor gene and loss of its control on Mitogen-Activated Protein Kinase (MAPK) activation. Mutation of NF1 is associated with decreased response to BRAF inhibitors and poor overall survival [39]. Mutations also occur in the proto-oncogene tyrosine-protein kinase KIT (c-KIT) in 1–7% of melanomas, being specifically associated to acral lentiginous melanoma and mucosal melanoma or chronic sun damage. Generally, major mutations (L576P and K642E) or copy number gains are present, resulting in increased MAPK signaling [40].

### 2.4. Lymph Node Evaluation

The sentinel lymph node status at diagnosis provides more accurate disease staging. The American Society of Clinical Oncology in the guidelines on the management of the regional lymph nodes in patients with melanoma declares that the sentinel lymph node biopsy is not recommended for patients with T1a melanoma, but may be considered for patients with T1b melanoma and it is recommended for melanomas classified T2–T4 [41]. Nodal (N) category indicates whether melanoma has moved from the primary site to the vicinal lymph nodes. This is a very important prognostic parameter indicating the spread of primary melanoma: the more lymph nodes involved, the higher the risk. In the AJCC evaluation, different subgroups are designated by 0 to 3, indicating the absence (N0) or presence of metastasis diffusion in only one (N1), in two or three (N2) and four or more lymph nodes (N3), respectively. NX indicates the absence of lymph node evaluation, while a second suffix, designated with a, b and c, specifies the presence of occult metastases in sentinel lymph node biopsy (indicated with a), or by physical or radiological detection in regional lymph nodes (indicated as b), as well as the presence of in-transit microsatellite metastases (indicated as c) [4].

### 2.5. New Possible Diagnostic Parameters

More recently, progress in the accuracy of melanoma diagnosis took advantage from the molecular approach to classify borderline lesions. These methods utilize DNA probe to measure cellular genetic copy numbers by fluorescent *in situ* hybridization (FISH) and single nucleotide polymorphism (SNP). Genome loci in different chromosomes have been evaluated evidencing chromosomal aberrations associated with different diseases, as abnormalities of chromosomes 5p, 11q, 12q and 15q in acral melanoma, and chromosomes 17p and 13q in lentigo maligna melanomas or severely sun-damaged skin. All these patterns of aberrations were nearly absent in benign nevi (96.2% aberrations in melanoma against 13% in nevi) [42,43].

## 3. Prognostic and Predictive Biomarkers

To complete the presentation of biomarkers for melanoma, categorization is essential to define a group of them capable to dissect the impact on tumor biology and disease course (prognostic biomarkers) and a second group defining tumor response to treatment (predictive biomarkers) as well as the improvement in overall survival (OS), disease free survival (DFS) and progression free survival (PFS). The last aspects are essential to define those patients that can have clinical benefit from therapeutic choice, particularly for immunotherapy that, if it has revolutionized the melanoma cure, still presents many questions to be addressed related to the limited efficacy and high toxicity [44]. To date, many possible biomarkers have been evaluated essentially in retrospective studies. Therefore, in the AJCC staging system, intrinsic limitations in the number of the categorized biomarkers were observed. Thus, many different parameters are normally utilized to obtain more accurate disease prognosis and prediction, although their implementation in larger data set is necessary to support a validation for routine procedures in clinical practice.

In primary melanoma, the prognosis is strongly dependent on tumor thickness, ulceration, mitotic rate, vasculature development, presence of tumor-infiltrating lymphocytes (TILs) and melanoma subtype. Other characteristics are intrinsic to the patients, such as age, sex and anatomical site of the lesion. In general, young and females are associated with a more favorable prognosis.

### 3.1. Genetic Prognostic Factors

The genetic background of a tumor, beyond addressing patients to specific treatment, has a clear prognostic value. BRAF mutation is the most common mutation of CM, generally affecting young people and characterized by decrease of CD8^+^ number and increased release of immunosuppressive cytokines. BRAF mutation displays unfavorable prognostic value compared to wild type melanomas, although may benefit of approved combination therapy with BRAF and MEK inhibitors (dabrafenib plus trametinib or vemurafenib plus cobimetinib). Unfortunately, these patients are often subjected to acquired resistance [45,46,47]. NRAS mutations identify a more aggressive disease and these mutations are predictor of poorer outcomes in view of the lack of any novel specific therapy, being progress limited to MEK inhibitors with modest clinical benefit in PFS and substantially inefficacious to increase the OS [48].

Other molecular markers with important prognostic and predictive value are guanine nucleotide-binding protein alpha subunits G(q) /11 (GNAQ/11) and TP53 mutations. GNAQ/11 are involved in signaling via G-protein-coupled receptors. GNAQ/11 mutations, found in uveal melanoma and in little percentage of the so-called triple wild type subtype of CM, induce overexpression of RAS Guanyl releasing protein 3 (RasGRP3) with consequent constitutive activation of RAS, event associated with poor OS. Currently, therapies for treatment of this mutation are essentially inadequate [49]. Associated with poor OS and PFS, the TP53 mutations are present in 20% of CM and characterized by senescence evasion and autophagy activation. TP53 mutations characterize older group of patients, mainly presenting head and neck melanomas [50]. Familial melanoma susceptibility is a sporadic event that occur in 2% of all melanomas and the incidence strongly increases with increased number of familiar cases. The screening among the familial population of the CDKN2A mutation is necessary and essential [51].

### 3.2. Prognostic Factors in Lymphangiogenesis

Melanoma cells secrete, among others, some vascular endothelial growth factors (VEGF), namely VEGF-C and -D that, together with their receptor VEGFR3, have been proposed as lymphangiogenetic markers, albeit with contradictory results [52]. Furthermore, lymphatic invasion is characterized by cancer cells ability to adhere to endothelial cells and to migrate along the lymphatic vasculature. In this regard, protein phosphatase 2 regulatory subunit A (PPP2R1A), responsible of active interaction between melanoma and lymphatic endothelial cells, has been proposed as a new biomarker in melanoma metastatization [53]. Overall, the immunohistochemical approach, although very expensive and time-consuming, provides a representative picture of the lymphohematological status of the primary tumor, yielding information for diagnosis and prognosis. In recent years, however, researchers have begun to look at lymph as a greater reservoir of cancer biomarkers compared to plasma liquid biopsy. In this respect, the use of postoperative lymphatic exudate from metastatic melanoma patients is becoming a powerful non-invasive clinical practice for the identification of tumor-derived factors, including extracellular vesicles charged of proteins and miRNAs reminiscent of metastatic progression [54,55].

### 3.3. Lymph Node Prognostic Role

Sentinel lymph node status was indicated as the major prognostic factor for disease free survival and choice of therapy in adjuvant setting [56]. Lymph nodal involvement correlated with tumor thickness and their biopsy was justified for thick melanomas (>4 mm), in presence of ulceration, tumor lymphocyte infiltration and mitotic rate >1 mm^2^. In the recent past, the possibility that patients with lymph nodal metastatic disease could benefit from complete surgical eradication of lymph nodal station/basin has generated a deep scientific debate that produced a number of studies [57]. Among these studies, two important international multicenter trials led to similar conclusions. In these trials, there appears to be no survival benefit associated with complete lymph node dissection, but for a major regional node control of the disease [58,59]. Consequently, surgical lymph node dissection for patients with sentinel lymph node-positive melanoma is no longer routinely recommended, mainly when melanoma had spread to distant sites (stage III and IV), also considering the major efficacy of targeted and immune strategies introduced in the adjuvant setting [60].

### 3.4. Prognostic and Predictive Biomarkers and Immunotherapy

**Figure 2:**
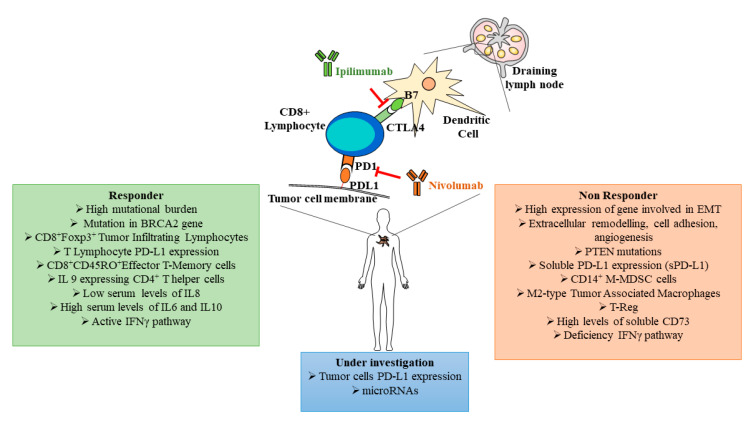
Schematic representation of some key immune checkpoint blocker functional links and predictive biomarkers associated to responder (green box) and non-responder (red box) patients. In the blue box are indicated melanoma biomarkers still under investigation. PD-L1: programed cell death ligand 1, PD-1: programed cell death 1, sPD-L1: soluble programed cell death ligand 1, CTLA4: cytotoxic T-lymphocyte associated protein 4, BRCA2: breast cancer type 2 susceptibility protein, Foxp3: forkhead box p3, IL: interleukin, IFN: interferon, EMT: epithelial–mesenchymal transition, PTEN: phosphatase and tensin homolog, MDSC: myeloid derived suppressor cells, M-MDSC: monocyte myeloid derived suppressor cells; T-Reg: regulatory T cells.

The recent use of ICBs as standard therapy for advanced melanomas evidenced efficacy only in a limited number of patients and adverse events, developed in some patients, were so severe to induce therapy blockage. Thus, it is now essential to characterize and validate standard biomarkers predicting response and toxicity before treatment, in order to achieve the best potential clinical benefit (Table 1, Figure 2). In melanoma, the high mutational burden correlates with more effective immunotherapy, considering that different somatic mutations can generate immunogenic tumor neoantigens able to sustain an immune response in ICB-responder melanoma patients [61]. Riaz and coworkers demonstrated that during immunotherapy with Nivolumab, compared to baseline, the tumor mutation burden decreased in responder patients. Furthermore, in these patients, the loss of a number of certain neoantigens proportionally reflexed the clonal expansion of T cell populations [62]. However, this association was able to predict improved survival but not treatment responses. In this respect, a transcriptomic study in biopsies of pretreated melanoma revealed that the mutational loads improved survival independently from the capacity to respond to immunotherapy, whereas responder patients were characterized by an enrichment for mutations in the DNA repair gene BRCA2. On the contrary, non-responder patients showed a transcriptional signature, referred as innate anti-programed cell death 1 (PD-1) resistance, with high expression of genes involved in regulation of epithelial mesenchymal transition, extracellular matrix remodeling, cell adhesion and angiogenesis (Figure 2) [61]. Several investigations found a direct correlation between gene expression associated to the interferon γ (IFNγ) pathway, T cell inflamed tumor microenvironment with abundant expression of chemokines and response to immune checkpoint blockers. Other studies indicated the deficiency of ICB clinical efficacy in melanomas characterized by PTEN mutations and functional deficiency of the IFNγ signaling triggered by loss-of-function mutations in Janus kinase JAK1/2 and β2 microglobulin (Table 1) [63,64,65]. 

Expression on tumor cells of programed cell death ligand 1 (PD-L1) has been evaluated as predictive of ICB response and considered a precondition for patient enrollment to immunotherapy. However, some divergences respect to this prerequisite emerged, indicating that the association of PD-L1 expression and the certainty of positive response to immunotherapy was unpredictable. Inflammatory state and treatments can regulate PD-L1 expression and its inconsistence as predictive biomarker was mainly evidenced by good response in patients with barely detectable PD-L1 expression [74]. A systematic review by Jessurun and coworkers provides an important overview of candidate predictive biomarkers for ICBs in melanoma patients [75]. In this study, the predictive value of PD-L1 was strongly influenced by its expression in different cell contexts. Thus, tumor PD-L1 expression can be considered a valid prognostic marker more than a predictive one. On the contrary, when PD-L1 expression level was considered in tumor immune cells population, its value as predictive biomarker to ICBs response was more consistent [76]. More specific is the negative predictive role of soluble PD-L1 on ICB response. High levels of sPD-L1 correlate with progression of advanced melanoma treated with ICBs showing incomplete response to treatment (Figure 2) [77].

Emerging data indicated the TIL population in tumor stroma as a predictive marker for response to immunotherapy and associated to a better OS. However, the significance of this presence produced contradictory information. Different meta-analyses demonstrated that the most adherent phenotype associated with favorable prognosis is the subpopulation characterized by coexpression of CD8/forkhead box p3 (FoxP3) compared to CD8 alone [44].

The effect of nivolumab was associated with reduced clonal expansion of the T cell receptor repertoire (TCR), characterized by loss of antigens during treatment and reflecting the decrease in tumor mutational load [62]. The association of mutational/neoantigen load and T cell clonality correlated with outcome associated with specific sequential ICB treatment: Yusko and coauthors demonstrated this association only when nivolumab was administered before ipilimumab, but not the opposite [78]. In general in melanoma, as in other solid tumors, the presence of CD14^+^ monocyte-myeloid derived suppressor (M-MDSC) cells, M2 tumor associated macrophages (M2-Type TAMs) and regulatory T cells in the tumor microenvironment and in blood circulation, negatively modulates the antitumor T lymphocyte activity, eventually favoring tumor growth and spreading, in turn largely reducing outcome of immunotherapy. The presence of all these regulatory cells should be considered and understood in detail, in association to treatment response [66,67,68]. In contrast, some studies indicated longer DFS and OS in response to ipilimumab treatment, which increased the proportion of circulating CD8^+^CD45RO^+^ effector memory T cells (Table 1, Figure 2) [69]. Considering how complex is the immune context in solid tumors, including melanoma, a comprehension of the mechanisms underlying the antitumor actions of different ICBs is necessary to characterize the effects on the immune profiles. The study of Wei and coworkers demonstrated two distinct cellular mechanisms underlying the anti-PD-1 and anti-cytotoxic T-lymphocyte associated protein 4 (CTLA-4) blockade immune response. Both the blockers induced the expansion of specific tumor-infiltrating exhausted-like CD8 T cell subsets, while only the anti-CTLA-4 treatment induced the robust expansion of the subset of inducible costimulatory^+^ (ICOS^+^) Th1-like CD4^+^ effector population [79]. Different studies on circulating CD4^+^ T cells revealed a strong association between good response to anti-PD-1 therapy and increased circulation of a subset of CD4^+^ memory cells, characterized by CD27^+^, FAS^−^, CD45RA^−^ and CCR7^+^ expression in association with interleukin 9 (IL-9) expressing CD4^+^ T helper cells (Table 1, Figure 2) [70]. Another interesting finding comes from studies on circulating CD8^+^ T cells expressing CD28 (a member of the same family of PD-1, CTLA-4 and ICOS), a molecule considered important in CD8 effector T cell inactivation following PD-L1 ligation. Hui and co-workers reported that PD-1 phosphorylation induced dephosphorylation of CD28 and inhibition of T cell proliferation. In lung cancer treated with immunotherapy, the rescue of exhausted CD8^+^ T cells was associated with expression of CD28 [80,81]. The validation of the described circulating immune cell subpopulations as prognostic and/or predictive factors of immunotherapy outcome needs some more studies before application in clinical setting.

Another important reservoir of possible biomarkers of immunotherapy response is represented by soluble plasma level of cytokines. Thus, the serum level of interleukin-8 was indicated as indicator of efficacy, reflecting tumor burden and response in melanoma patients treated with nivolumab plus ipilimumab. In these patients, the responder group showed decreased levels of IL-8 from baseline to the best response with ICBs and early decrease was associated with longer overall survival (Figure 2). Furthermore, VEGF-C level correlated with T cell expansion and potentiated response to ICBs [71,82]. In murine model of melanoma, it has been demonstrated that VEGF-C was able to promote immune response via induction of CCL21 and tumor infiltration of naïve T cells before immunotherapy, indicating serum VEGF-C as a predictive biomarker for immunotherapy response [82]. In parallel, multivariate analysis of human melanoma specimens demonstrated VEGF-C as an independent predictor of metastatic risk [83].

Among patients treated with nivolumab, higher serum levels of IL-6, INFγ and IL-10 before treatment were associated to responder patients, while elevated basal levels of soluble CD73 (sCD73), the enzyme that hydrolyzes the extracellular adenosine monophosphate (AMP) into adenosine, were associated with lower response rate to treatment (Table 1, Figure 2) [72,73].

The prognostic role of eosinophil count was evaluated in retrospective studies. In melanoma patients, treated or not with ICBs, the eosinophilic count was associated with longer survival independently from therapy. Indeed, in patients treated with immunotherapy, survival improved proportionally to eosinophilia level [84].

### 3.5. miRNAs as Possible Biomarkers

Among new possible biomarkers in melanoma, circulating microRNAs (miRNAs) have always attracted many attentions for the enormous potential of correlating their expression with different phases of tumor disease. More than 2600 miRNAs are encoded by human genome and their biogenesis has been largely described [85,86]. Implicated in numerous cellular functions, the higher expression of miRNAs with an oncogenic role can favor tumor development and progression, acting on proliferation, migration and metastatization of tumor cells. Conversely, other miRNAs with tumor suppressive function are systematically silenced [87]. Their potential as non-invasive biomarkers are associated with their release in circulation as free molecules, included in microvesicles and exosomes or linked to lipoproteins. In this way, miRNAs are protected and transported in the blood to distant districts, where can be released establishing remote communication between cells [88]. Generally, the function associated with their release is protumoral and contribute to remodel tumor microenvironment or metastatic distant sites to favor tumor growth and tumor cell colonization, respectively. The high stability of exosomes or the protective role of lipoproteins, that rescue miRNAs from degradation in the body fluids, make advantageous their use as biomarker with diagnostic and prognostic utility in different cancer type, including melanoma [89,90]. Efforts to define in melanoma miRNAs with diagnostic value have been proposed by reports that demonstrated different expression profiles in serum of patients compared to healthy people. Unfortunately, divergence in sample source (plasma or serum), methodology, data set generation and interpretation made validation of these results very difficult [91,92]. More intriguing is the potential of using miRNAs as prognostic biomarkers, assisting the clinicians to distinguish high recurrent disease before metastasis development. We are at beginning of the journey and associating validated prognostic and/or predictive roles to specific miRNA signatures is challenging due to limitations on sample collection, processing and analysis (Figure 2) [93]. A good example of the tortuous journey to validate the prognostic role of miRNAs comes from miR-16 study. This miRNA has been shown to be gradually downregulated from healthy, stage I/II and during progression to III/IV stage, suggesting both diagnostic and prognostic value, as its expression positively correlated with longer survival [94]. On the contrary, previous studies demonstrated that miR-16 levels increased in melanoma patients during progression and in healthy people its expression was positively influenced by stress [95,96]. Therefore, these contradictory results indicated that miR-16 could not be used as melanoma biomarker, confirming how difficult is to definitely validate a biomarker.

## 4. Conclusions

In the last decade, clinical studies have shown important improvements in patients with metastatic melanoma, the first cancer successfully treated with ICB. Now an important added value should be the capability to choose the more promising first-line therapeutic approach, such us selecting targeted or ICB therapies in BRAF mutated melanomas. In addition, the identification of predictive biomarkers able to select responders from non-responders and, possibly, progression from long-lasting disease-free people would represent a fundamental additional step. Moreover, in the presence of complete remissions, the capability to continue or stop the treatments without increasing the risk of recurrence will give us the opportunity to reduce the adverse events associated with very long treatments.

To this end, new approaches are under study. One of this is the RNA-Seq technique, able to analyze the whole transcriptome in the plasma or serum of patients, offering higher quantification and detection of poor abundant mRNA transcripts. Besides miRNAs, this analysis includes the expression of other regulatory RNA molecules, such as small nucleolar RNAs, long non-coding RNAs, piwi-interacting RNA and transfer RNAs, all interesting as new possible biomarkers for a better melanoma prognosis and prediction. Another aspect that is becoming relevant is the role of microbiome in view of its complex crosstalk with the immune system. Indeed, patients with an “unfavorable” gut microbiome showed reduced antitumor immune responses because of limited intratumoral lymphoid infiltrated and reduced antigen presentation capacity [97].

All together, these results indicate that, although significant progress has been obtained in the last few years, we have still to add some pieces to this complex puzzle to actually move from evidence-based to a precision medicine that, taking account of algorithms, will give us the possibility of selecting the best therapeutic approach for each person.

## Figures and Tables

**Table 1:** Activity of predictive cellular and circulating biomarkers described in Figure 2. sCD73: soluble CD73, IFN: interferon, IL: interleukin, T-reg: regulatory T cell, M2-type TAMs: M2 tumor associated macrophages, M-MDSCs: monocyte-myeloid derived suppressor cells, Th-cells: T helper cells, TILs: tumor-infiltrating lymphocytes.

**Cellular Markers**	**Activity**	**References**
CD8^+^Foxp3^+^ TILs	Antitumoral acitivity	[44]
CD8^+^ T Cytotoxic cells	Antigen primed cells against tumor cells, encountering dysfunction and exhaustion due to immunosuppression	[46]
CD14^+^ M-MDSCs	Cell population that inhibits T cell activation	[66]
M2-Type TAMs	Negatively modulates the antitumor T lymphocyte activity	[67]
T-reg	Cells that produce cytokines with immunosuppressive activities	[68]
CD8^+^CD45RO^+^ cells	T Memory cell subset	[69]
CD4^+^ Th-cells	Secreting cytokines with differential activities on other immune system cells	[70]
**Circulating Molecules**	**Activity**	**References**
IL-9	Anti-tumoral actions in melanoma, increases granzyme B and perforin in CD8^+^ T cells	[70]
IL-8	Key neutrophil chemotactic factor inducing chemotaxis and phagocytosis of target cells	[71]
IL-6	Key pleiotropic cytokine with pro-tumorigenic and anti-tumoral role	[72]
IL-10	Key immune-suppressive cytokine produced by T-reg	[72]
IFNγ	Cytokine playing an important role in inducing and modulating an array of immune responses	[72]
sCD73	Participates in the extracellular production of adenosine that down-regulates inflammatory and immune responses	[73]

## References

[1] Matthews N.H., Li W.Q., Qureshi A.A., Weinstock M.A., Cho E., Ward W.H., Farma J.M. (2017). Epidemiology of Melanoma. Cutaneous Melanoma: Etiology and Therapy.

[2] Amin M.B., Edge S., Greene F., Byrd D.R., Brookland R.K., Washington M.K., Gershenwald J.E., Compton C.C., Hess K.R., Sullivan D.C. (2017). AJCC Cancer Staging Manual.

[3] Tracey E.H., Vij A. (2019). Updates in Melanoma. Dermatol. Clin..

[4] Gershenwald J.E., Scolyer R.A., Hess K.R., Sondak V.K., Long G.V., Ross M.I., Lazar A.J., Faries M.B., Kirkwood J.M., McArthur G.A. (2017). Melanoma staging: Evidence-based changes in the American Joint Committee on Cancer eighth edition cancer staging manual. CA Cancer J. Clin..

[5] Donnelly D., Aung P.P., Jour G. (2019). The “-OMICS” facet of melanoma: Heterogeneity of genomic, proteomic and metabolomic biomarkers. Semin. Cancer Biol..

[6] Arozarena I., Wellbrock C. (2017). Targeting invasive properties of melanoma cells. FEBS J..

[7] American Cancer Society (2020). Cancer Facts & Figures 2020.

[8] Ferlay J., Colombet M., Soerjomataram I., Mathers C., Parkin D.M., Pineros M., Znaor A., Bray F. (2019). Estimating the global cancer incidence and mortality in 2018: GLOBOCAN sources and methods. Int. J. Cancer.

[9] Schwartz M.R., Luo L., Berwick M. (2019). Sex Differences in Melanoma. Curr. Epidemiol. Rep..

[10] Courtenay W. (2000). Behavioral Factors Associated with Disease, Injury, and Death among Men: Evidence and Implications for Prevention. J. Men Stud..

[11] Paddock L.E., Lu S.E., Bandera E.V., Rhoads G.G., Fine J., Paine S., Barnhill R., Berwick M. (2016). Skin self-examination and long-term melanoma survival. Melanoma Res..

[12] Joosse A., de Vries E., Eckel R., Nijsten T., Eggermont A.M., Hölzel D., Coebergh J.W.W., Engel J. (2011). Gender Differences in Melanoma Survival: Female Patients Have a Decreased Risk of Metastasis. J. Investig. Dermatol..

[13] Gupta S., Artomov M., Goggins W., Daly M., Tsao H. (2015). Gender Disparity and Mutation Burden in Metastatic Melanoma. J. Natl. Cancer Inst..

[14] Nonaka D., Chiriboga L., Rubin B.P. (2008). Differential expression of S100 protein subtypes in malignant melanoma, and benign and malignant peripheral nerve sheath tumors. J. Cutan. Pathol..

[15] Ribé A., McNutt N.S., Rib A. (2003). S100A6 Protein Expression is Different in Spitz Nevi and Melanomas. Mod. Pathol..

[16] Eisenstein A., Gonzalez E.C., Raghunathan R., Xu X., Wu M., McLean E.O., McGee J., Ryu B., Alani R.M. (2018). Emerging Biomarkers in Cutaneous Melanoma. Mol. Diagn. Ther..

[17] Weinstein D., Leininger J., Hamby C., Safai B. (2014). Diagnostic and Prognostic Biomarkers in Melanoma. J. Clin. Aesthet. Dermatol..

[18] Ordóñez N.G. (2014). Value of melanocytic associated immunohistochemical markers in the diagnosis of malignant melanoma: A review and update. Hum. Pathol..

[19] Salazar-Onfray F., López M., Lundqvist A., Aguirre A., Escobar A., Serrano A., Korenblit C., Petersson M., Chhajlani V., Larsson O. (2002). Tissue distribution and differential expression of melanocortin 1 receptor, a malignant melanoma marker. Br. J. Cancer.

[20] Blochin E., Nonaka D. (2009). Diagnostic value of Sox10 immunohistochemical staining for the detection of metastatic melanoma in sentinel lymph nodes. Histopathology.

[21] Abbas O., Miller D.D., Bhawan J. (2014). Cutaneous malignant melanoma: Update on diagnostic and prognostic biomarkers. Am. J. Dermatopathol..

[22] Xu X., Gimotty P.A., Guerry D., Karakousis G., Elder D.E. (2014). Lymphatic invasion as a prognostic biomarker in primary cutaneous melanoma. Methods Mol. Biol..

[23] Špirić Z., Erić M., Eri Ž. (2017). Lymphatic invasion and the Shields index in predicting melanoma metastases. J. Plast. Reconstr. Aesthet. Surg..

[24] Banerji S., Ni J., Wang S.-X., Clasper S., Su J., Tammi R., Jones M., Jackson D.G. (1999). LYVE-1, a New Homologue of the CD44 Glycoprotein, Is a Lymph-specific Receptor for Hyaluronan. J. Cell Biol..

[25] Kahn H.J., Marks A. (2002). A New Monoclonal Antibody, D2-40, for Detection of Lymphatic Invasion in Primary Tumors. Lab. Investig..

[26] Špirić Z., Vještica M., Erić M. (2020). Survival prediction in patients with cutaneous melanoma by tumour lymphangiogenesis. Acta Clin. Belg..

[27] Bradish J.R., Cheng L. (2014). Molecular pathology of malignant melanoma: Changing the clinical practice paradigm toward a personalized approach. Human Pathol..

[28] Long G.V., Menzies A.M., Nagrial A.M., Haydu L.E., Hamilton A.L., Mann G., Hughes T.M., Thompson J.F., Scolyer R.A., Kefford R. (2011). Prognostic and Clinicopathologic Associations of Oncogenic BRAF in Metastatic Melanoma. J. Clin. Oncol..

[29] Hugdahl E., Kalvenes M.B., Puntervoll H.E., Ladstein R.G., Akslen L.A. (2016). BRAF-V600E ex-pression in primary nodular melanoma is associated with aggressive tumour features and re-duced survival. Br. J. Cancer.

[30] Bos J.L. (1989). Ras oncogenes in human cancer: A review. Cancer Res..

[31] Devitt B., Liu W., Salemi R., Wolfe R., Kelly J., Tzen C.Y., Dobrovic A., McArthur G. (2011). Clinical outcome and pathological features associated with NRAS mutation in cutaneous melanoma. Pigment Cell Melanoma Res..

[32] Curtin J., Fridlyand J., Kageshita T., Patel H.N., Busam K.J., Kutzner H., Cho K.-H., Aiba S., Bröcker E.-B., LeBoit P.E. (2005). Distinct Sets of Genetic Alterations in Melanoma. N. Engl. J. Med..

[33] Tsao H., Bevona C., Goggins W., Quinn T. (2003). The transformation rate of moles (melanocytic nevi) into cutaneous melanoma: A population-based estimate. Arch. Dermatol..

[34] Psaty E.L., Scope A., Halpern A.C., Marghoob A.A. (2010). Defining the patient at high risk for melanoma. Int. J. Dermatol..

[35] Gumaste P.V., Penn L.A., Cymerman R.M., Kirchhoff T., Polsky D., McLellan B. (2015). Skin cancer risk in BRCA1/2 mutation carriers. Br. J. Dermatol..

[36] Garibyan L., Fisher D.E. (2010). How Sunlight Causes Melanoma. Curr. Oncol. Rep..

[37] Miller A.J., Mihm M.C. (2006). Melanoma. N. Engl. J. Med..

[38] Guterres A., Herlyn M., Villanueva J. (2018). Melanoma. eLs.

[39] Cirenajwis H., Lauss M., Ekedahl H., Törngren T., Kvist A., Saal L.H., Olsson H., Staaf J., Carneiro A., Ingvar C. (2017). NF1-mutated melanoma tumors harbor distinct clinical and biological characteristics. Mol. Oncol..

[40] Beadling C., Jacobson-Dunlop E., Hodi F.S., Le C., Warrick A., Patterson J., Town A., Harlow A., Cruz F., Azar S. (2008). KIT gene mutations and copy number in melanoma sub-types. Clin. Cancer Res..

[41] Lange J.R. (2020). Review of the Guidelines on the Management of the Regional Lymph Nodes in Patients with Melanoma. JAMA Surg..

[42] Foster J.M., Oumie A., Togneri F.S., Vasques F.R., Hau D., Taylor M., Tinkler-Hundal E., Southward K., Medlow P., McGreeghan-Crosby K. (2015). Cross-laboratory validation of the OncoScan^®^ FFPE Assay, a multiplex tool for whole genome tumour profiling. BMC Med. Genom..

[43] Bastian B.C., Olshen A.B., LeBoit P.E., Pinkel D. (2003). Classifying Melanocytic Tumors Based on DNA Copy Number Changes. Am. J. Pathol..

[44] Kitano S., Nakayama T., Yamashita M. (2018). Biomarkers for Immune Checkpoint Inhibitors in Melanoma. Front. Oncol..

[45] Ascierto P.A., McArthur G.A., Dréno B., Atkinson V., Liszkay G., Di Giacomo A.M., Mandalà M., Demidov L., Stroyakovskiy D., Thomas L. (2016). Cobimetinib combined with vemu-rafenib in advanced BRAF(V600)-mutant melanoma (coBRIM): Updated efficacy results from a randomised, double-blind, phase 3 trial. Lancet Oncol..

[46] Ugurel J., Rohmel P.A., Ascierto K.T., Flaherty J.J., Grob A., Hauschild J., Larkin G.V., Long P., Lorigan G.A., McArthur A. (2016). Survival of patients with advanced metastatic melanoma: The impact of novel therapies. Eur. J. Cancer.

[47] Fujimura T., Hidaka T., Kambayashi Y., Aiba S. (2018). BRAF kinase inhibitors for treatment of melanoma: Developments from early-stage animal studies to Phase II clinical trials. Expert Opin. Investig. Drugs.

[48] Sarkisian S., Davar D. (2018). MEK inhibitors for the treatment of NRAS mutant melanoma. Drug Des. Dev. Ther..

[49] Livingstone E., Zaremba A., Horn S., Ugurel S., Casalini B., Schlaak M., Hassel J.C., Herbst R., Utikal J.S., Weide B. (2020). GNAQ and GNA11 mutant nonuveal melanoma: A sub-type distinct from both cutaneous and uveal melanoma. Br. J. Dermatol..

[50] Viros A., Sanchez-Laorden B., Pedersen M., Furney S.J., Rae J., Hogan K., Ejiama S., Girotti M.R., Cook M., Dhomen N. (2014). Ultraviolet radiation accelerates BRAF-driven melanomagenesis by targeting TP53. Nature.

[51] Zebary A., Omholt K., van Doorn R., Ghiorzo P., Harbst K., Johansson C.H., Höiom V., Jönsson G., Pjanova D., Puig S. (2014). Somatic BRAF and NRAS mutations in familial melanomas with known germline CDKN2A status: A GenoMEL study. J. Investig. Dermatol..

[52] Pasquali S., van der Ploeg A.P., Mocellin S., Stretch J.R., Thompson J.F., Scolyer R.A. (2013). Lymphatic biomarkers in primary melanomas as predictors of regional lymph node metastasis and patient outcomes. Pigment Cell Melanoma Res..

[53] Christianson D.R., Dobroff A.S., Proneth B., Zurita A.J., Salameh A., Dondossola E., Makino J., Bologa C.G., Smith T.L., Makino J. (2015). Ligand-directed targeting of lymphatic vessels uncovers mechanistic insights in melanoma metastasis. Proc. Natl. Acad. Sci. USA.

[54] Broggi M.A., Maillat L., Clement C.C., Bordry N., Corthésy P., Auger A., Matter M., Hamelin R., Potin L., Demurtas D. (2019). Tumor-associated factors are enriched in lymphatic exudate compared to plasma in metastatic melanoma patients. J. Exp. Med..

[55] García-Silva S., Benito-Martín A., Sánchez-Redondo S., Hernández-Barranco A., Ximénez-Embún P., Nogués L., Mazariegos M.S., Brinkmann K., López A.A., Meyer L. (2019). Use of extracellular vesicles from lymphatic drainage as surrogate markers of melanoma progression and BRAFV600E mutation. J. Exp. Med..

[56] Gershenwald J.E., Thompson W., Mansfield P.F., Lee J.E., Colome M.I., Tseng C.H., Lee J.J., Balch C.M., Reintgen D.S., Ross M.I. (1999). Multi-institutional melanoma lymphatic mapping experience: The prognostic value of sentinel lymph node status in 612 stage I or II melanoma patients. J. Clin. Oncol..

[57] Gorantla V.C., Kirkwood J.M. (2014). State of melanoma: An historic overview of a field in transition. Hematol. Oncol. Clin. N. Am..

[58] Faries M.B., Thompson J.F., Cochran A.J., Andtbacka R.H., Mozzillo N., Zager J.S., Jahkola T., Bowles T.L., Testori A., Beitsch P.D. (2017). Completion Dissection or Observation for Sentinel-Node Metastasis in Melanoma. N. Engl. J. Med..

[59] Leiter U., Stadler R., Mauch C., Hohenberger W., Brockmeyer N., Berking C., Sunderkötter C., Kaatz M., Schulte K.W., Lehmann P. (2016). Complete lymph node dissection versus no dissection in patients with sentinel lymph node biopsy positive melanoma (DeCOG-SLT): A multicentre, randomised, phase 3 trial. Lancet Oncol..

[60] Ascierto P.A., Borgognoni L., Botti G., Guida M., Marchetti P., Mocellin S., Muto P., Palmieri G., Patuzzo R., Quaglino P. (2019). New paradigm for stage III melanoma: From surgery to adjuvant treatment. J. Transl. Med..

[61] Hugo W., Zaretsky J.M., Sun L., Song C., Moreno B.H., Hu-Lieskovan S., Berent-Maoz B., Pang J., Chmielowski B., Cherry G. (2016). Genomic and Transcriptomic Features of Response to Anti-PD-1 Therapy in Metastatic Melanoma. Cell.

[62] Riaz N., Havel J., Makarov V., Desrichard A., Urba W.J., Sims J.S., Hodi F.S., Martín-Algarra S., Mandal R., Sharfman W.H. (2017). Tumor and Microenvironment Evolution during Immunotherapy with Nivolumab. Cell.

[63] Peng W., Chen J.Q., Liu C., Malu S., Creasy C., Tetzlaff M.T., Xu C., McKenzie J.A., Zhang C., Liang X. (2016). Loss of PTEN Promotes Resistance to T Cell-Mediated Immunotherapy. Cancer Discov..

[64] Zaretsky J.M., Garcia-Diaz A., Shin D.S., Escuin-Ordinas H., Hugo W., Hu-Lieskovan S., Torrejon D.Y., Abril-Rodriguez G., Sandoval S., Barthly L. (2016). Mutations Associated with Acquired Resistance to PD-1 Blockade in Melanoma. N. Engl. J. Med..

[65] Shin D.S., Zaretsky J.M., Escuin-Ordinas H., Garcia-Diaz A., Hu-Lieskovan S., Kalbasi A., Grasso C.S., Hugo W., Sandoval S., Torrejon D.Y. (2017). Primary Resistance to PD-1 Blockade Mediated by JAK1/2 Mutations. Cancer Discov..

[66] Kumar V., Patel S., Tcyganov E., Gabrilovich D.I. (2016). The Nature of Myeloid-Derived Suppressor Cells in the Tumor Microenvironment. Trends Immunol..

[67] Pollard J.W. (2004). Tumour-educated macrophages promote tumour progression and metastasis. Nat. Rev. Cancer.

[68] Ouyang Z., Wu H., Li L., Luo Y., Li X., Huang G. (2016). Regulatory T cells in the immunotherapy of melanoma. Tumor Biol..

[69] Tietze J.K., Angelova D., Heppt M.V., Reinholz M., Murphy W.J., Spannagl M., Ruzicka T., Berking C. (2017). The proportion of circulating CD45RO^+^ CD8^+^ memory T cells is correlated with clinical response in melanoma patients treated with ipilimumab. Eur. J. Cancer.

[70] Nonomura Y., Otsuka A., Nakashima C., Seidel J., Kitoh A., Dainichi T., Nakajima S., Sawada Y., Matsushita S., Aoki M. (2016). Peripheral blood Th9 cells are a possible pharmacodynamic biomarker of nivolumab treatment efficacy in metastatic melanoma patients. OncoImmunology.

[71] Sanmamed M.F., Perez-Gracia J.L., Schalper K.A., Fusco J.P., Gonzalez A., Rodriguez-Ruiz M.E., Oñate C., Perez G., Alfaro C., Martín-Algarra S. (2017). Changes in serum interleukin-8 (IL-8) levels reflect and predict response to anti-PD-1 treatment in melanoma and non-small-cell lung cancer patients. Ann. Oncol..

[72] Yamazaki N., Kiyohara Y., Uhara H., Iizuka H., Uehara J., Otsuka F., Fujisawa Y., Takenouchi T., Isei T., Iwatsuki K. (2017). Cytokine biomarkers to predict antitumor responses to nivolumab suggested in a phase 2 study for advanced melanoma. Cancer Sci..

[73] Morello S., Capone M., Sorrentino C., Giannarelli D., Madonna G., Mallardo D., Grimaldi A.M., Pinto A., Ascierto P.A. (2017). Soluble CD73 as biomarker in patients with metastatic melanoma patients treated with nivolumab. J. Transl. Med..

[74] Hutarew G. (2016). PD-L1 testing, fit for routine evaluation? From a pathologist’s point of view. Memo Mag. Eur. Med. Oncol..

[75] Jessurun C.A.C., Vos J.A.M., Limpens J., Luiten R.M. (2017). Biomarkers for Response of Melanoma Patients to Immune Checkpoint Inhibitors: A Systematic Review. Front. Oncol..

[76] Herbst R.S., Soria J.-C., Kowanetz M., Fine G.D., Hamid O., Gordon M.S., Sosman J.A., McDermott D.F., Powderly J.D., Gettinger S.N. (2014). Predictive correlates of response to the anti-PD-L1 antibody MPDL3280A in cancer patients. Nat. Cell Biol..

[77] Zhou J., Mahoney K.M., Giobbie-Hurder A., Zhao F., Lee S., Liao X., Rodig S., Li J., Wu X., Butterfield L.H. (2017). Soluble PD-L1 as a Biomarker in Malignant Melanoma Treated with Checkpoint Blockade. Cancer Immunol. Res..

[78] Yusko E., Vignali M., Wilson R.K., Mardis E.R., Hodi F.S., Horak C., Chang H., Woods D.M., Robins H., Weber J. (2019). Association of Tumor Microenvironment T-cell Repertoire and Mutational Load with Clinical Outcome after Sequential Checkpoint Blockade in Melanoma. Cancer Immunol. Res..

[79] Wei S.C., Levine J.H., Cogdill A.P., Zhao Y., Anang N.A.S., Andrews M.C., Sharma P., Wang J., Wargo J.A., Pe’er D. (2017). Distinct cellular mechanisms underlie anti-CTLA-4 and an-ti-PD-1 check-point blockade. Cell.

[80] Hui E., Cheung J., Zhu J., Su X., Taylor M.J., Wallweber H.A., Sasmal D.K., Huang J., Kim J.M., Mellman I. (2017). T cell costimulatory receptor CD28 is a primary target for PD-1–mediated inhibition. Science.

[81] Kamphorst A.O., Wieland A., Nasti T., Yang S., Zhang R., Barber D.L., Konieczny B.T., Daugherty C.Z., Koenig L., Yu K. (2017). Rescue of exhausted CD8 T cells by PD-1-targeted therapies is CD28-dependent. Science.

[82] Fankhauser M., Broggi M.A.S., Potin L., Bordry N., Jeanbart L., Lund A.W., Da Costa E., Hauert S., Rincon-Restrepo M., Tremblay C. (2017). Tumor lymphangiogenesis promotes T cell infiltration and potentiates immunotherapy in melanoma. Sci. Transl. Med..

[83] Špirić Z., Eri Ž., Erić M. (2017). Lymphatic vessel density and VEGF-C expression as independent predictors of melanoma metastases. J. Plast. Reconstr. Aesthet. Surg..

[84] Moreira A., Leisgang W., Schuler G., Heinzerling L. (2017). Eosinophilic count as a biomarker for prognosis of melanoma patients and its importance in the response to immunotherapy. Immunotherapy.

[85] Han J., Lee Y., Yeom K.-H., Kim Y.K., Jin H., Kim V.N. (2004). The Drosha-DGCR8 complex in primary microRNA processing. Genes Dev..

[86] Chendrimada T.P., Gregory R.I., Kumaraswamy E., Norman J., Cooch N., Nishikura K., Shiekhattar R. (2005). TRBP recruits the Dicer complex to Ago2 for microRNA processing and gene silencing. Nature.

[87] Hanahan D., Weinberg R.A. (2011). Hallmarks of Cancer: The Next Generation. Cell.

[88] Pontecorvi G., Bellenghi M., Puglisi R., Carè A., Mattia G. (2020). Tumor-derived extracellular vesicles and microRNAs: Functional roles, diagnostic, prognostic and therapeutic options. Cytokine Growth Factor Rev..

[89] Valadi H., Ekstrom K., Bossios A., Sjostrand M., Lee J.J., Lotvall J.O. (2007). Exosome-mediated transfer of mRNAs and microRNAs is a novel mechanism of genetic exchange between cells. Nat. Cell Biol..

[90] Vickers K.C., Palmisano B.T., Shoucri B.M., Shamburek R.D., Remaley A.T. (2011). MicroRNAs are transported in plasma and delivered to recipient cells by high-density lipoproteins. Nat. Cell Biol..

[91] Leidinger P., Keller A., Borries A., Reichrath J., Rass K., Jager S.U., Lenhof H.-P., Meese E. (2010). High-throughput miRNA profiling of human melanoma blood samples. BMC Cancer.

[92] Van Laar R., Lincoln M., Van Laar B. (2018). Development and validation of a plasma-based melanoma biomarker suitable for clinical use. Br. J. Cancer.

[93] Mumford S.L., Towler B.P., Pashler A.L., Gilleard O., Martin Y., Newbury S.F. (2018). Circulating MicroRNA Biomarkers in Melanoma: Tools and Challenges in Personalised Medicine. Biomolecules.

[94] Guo S., Guo W., Li S., Dai W., Zhang N., Zhao T., Wang H., Ma J., Yi X., Ge R. (2016). Se-rum miR-16: A Potential Biomarker for Predicting Melanoma Prognosis. J. Investig. Dermatol..

[95] Stark M.S., Klein K., Weide B., Haydu L.E., Pflugfelder A., Tang Y.H., Palmer J.M., Whiteman D.C., Scolyer R.A., Mann G.J. (2015). The Prognostic and Predictive Value of Melanoma-related MicroRNAs Using Tissue and Serum: A MicroRNA Expression Analysis. EBio Med..

[96] Katsuura S., Kuwano Y., Yamagishi N., Kurokawa K., Kajita K., Akaike Y., Nishida K., Masuda K., Tanahashi T., Rokutan K. (2012). MicroRNAs miR-144/144* and miR-16 in peripheral blood are potential biomarkers for naturalistic stress in healthy Japanese medical students. Neurosci. Lett..

[97] Gopalakrishnan V., Spencer C.N., Nezi L., Reuben A., Andrews M.C., Karpinets T.V., Prieto P.A., Vicente D., Hoffman K., Wei S.C. (2018). Gut microbiome modulates response to anti–PD-1 immunotherapy in melanoma patients. Science.

